# Successful use of a prophylactic cerebral protection device via the radial artery during left atrial appendage occlusion device implantation in a patient with a mitral valve mass

**DOI:** 10.1016/j.hrcr.2021.01.009

**Published:** 2021-01-26

**Authors:** Kunal Shah, Ryan R. Reeves, Anna Narezkina, Luis R. Castellanos, Frederick T. Han, Jonathan C. Hsu

**Affiliations:** Division of Cardiovascular Medicine, Sulpizio Cardiovascular Center, University of California San Diego, La Jolla, California

**Keywords:** Atrial fibrillation, Cerebral protection device, Mitral valve mass, SENTINEL device, Thrombus, WATCHMAN device

## Introduction

Stroke remains a debilitating complication from atrial fibrillation (AF), and AF-related strokes are responsible for over 15% of all stroke cases in the United States.^1^^,^^2^ While systemic oral anticoagulation can mitigate the risk of stroke, many patients cannot tolerate these medications. Percutaneous left atrial appendage occlusion (LAAO) is an alternative to oral anticoagulation for stroke prophylaxis in AF patients who cannot tolerate these medications long-term. In the United States, the WATCHMAN^TM^ (Boston Scientific, Marlborough, MA) for LAAO is the only device currently approved by the United States Food and Drug Administration for this indication.^3^, ^4^, ^5^

The presence of a left atrial appendage (LAA) thrombus is a contraindication to WATCHMAN insertion; however, there is little known regarding management of patients with intracardiac thrombi or masses in other locations. We present a rare case of WATCHMAN insertion in the setting of a chronic mitral valve mass and prophylactic use of a cerebral embolic protection device, the SENTINEL^TM^ (Boston Scientific, Santa Rosa, CA), to prevent stroke. The SENTINEL device was studied in the transcatheter aortic valve replacement (TAVR) population and shown to safely capture debris dislodged during the procedure in 99% of patients.^6^ Subsequent follow-up studies demonstrate that the SENTINEL device may be able to reduce the risk of stroke and mortality at 30 days post TAVR.^7^^,^^8^ Using this pre-existing experience with SENTINEL, we aimed to translate this effect to prevent cerebral embolization during WATCHMAN insertion.

## Case report

A 77-year-old man was admitted to our quaternary healthcare facility for an elective percutaneous LAAO procedure with a WATCHMAN device and planned temporary prophylactic SENTINEL device placement for cerebral embolism protection.

### Past medical history

The patient had a history of paroxysmal AF, chronic mass of the mitral valve, sick sinus syndrome with dual-chamber pacemaker implantation, recurrent syncope and falls, coronary artery disease with drug eluting stent in the left anterior descending artery, heart failure with preserved ejection fraction, prior transient ischemic attack, multiple myeloma, diabetes mellitus, hypertension, and chronic kidney disease. During admissions for syncope, the clinical team believed the cause was a combination vasovagal syndrome and AF with rapid ventricular response. Atrioventricular block was less likely given that his pacemaker was implanted prior to these episodes. In addition, a magnetic resonance angiography of the head and neck ruled out vertebrobasilar insufficiency.

### Differential diagnosis

The differential diagnosis for the mitral valve mass includes papillary fibroelastoma, myxoma, chronic thrombus, chronic vegetation, and mitral annular calcification.

### Investigations

**Figure 1:**
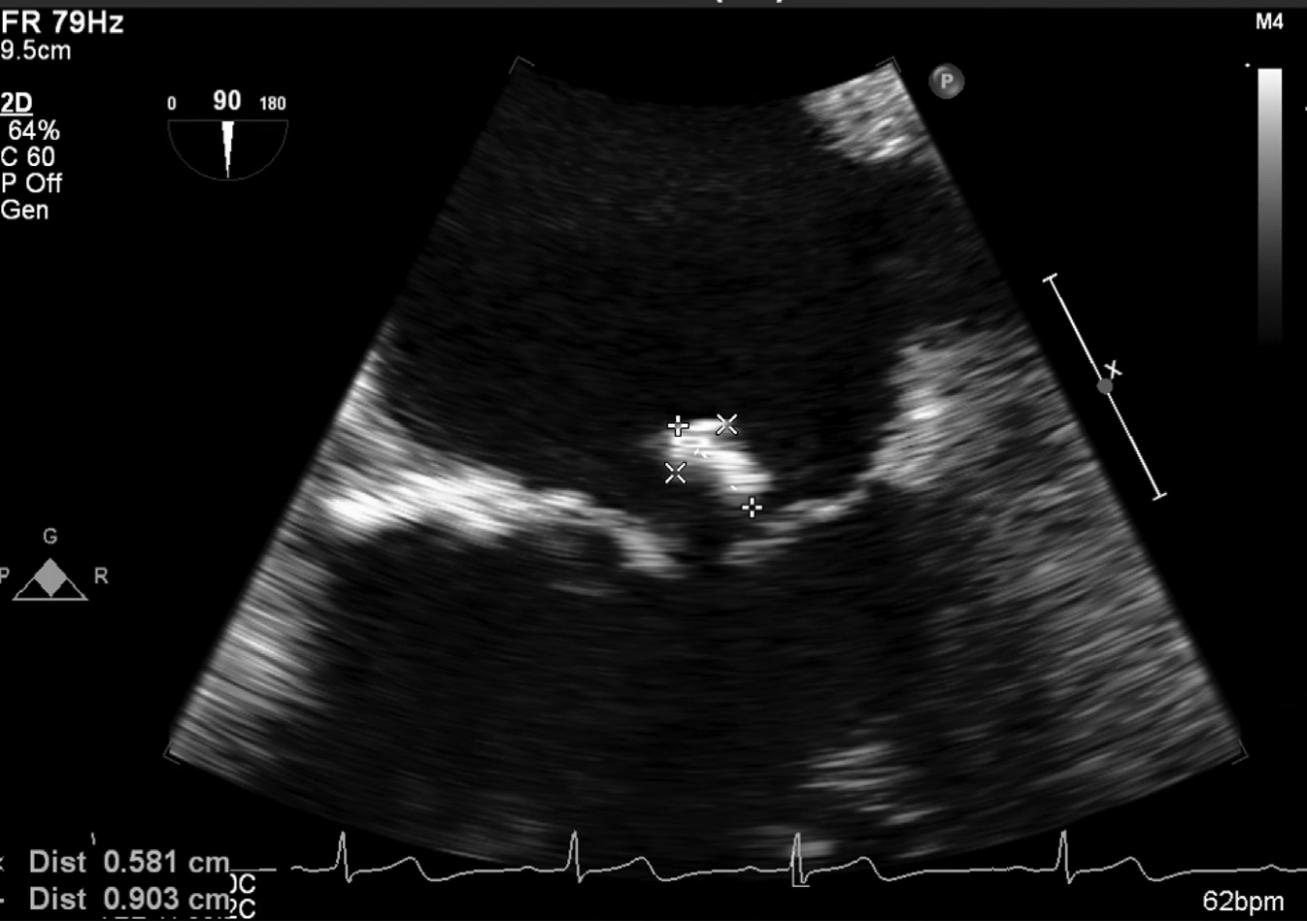
Preoperative image demonstrating a mitral valve mass. Transesophageal echocardiogram image at 90° showing size of mass attached to the posterior leaflet prior to WATCHMAN (Boston Scientific, Marlborough, MA) insertion.

The patient was considered for oral anticoagulation for stroke prevention given his significantly elevated CHA_2_DS_2_-VASc score of 8 (age greater than 75, diabetes mellitus, hypertension, coronary artery disease, heart failure, and transient ischemic attack). However, the presence of recurrent syncope, falls, and head trauma placed him at a high bleeding risk in the long term and he was never started on a direct anticoagulant. His HAS-BLED score was 5 (age greater than 65, hypertension, renal disease, clopidogrel usage, and prior transient ischemic attack) and he was therefore referred for percutaneous LAAO. The echodensity of the mitral valve was discovered incidentally on an echocardiogram 2 years prior to presentation in 2017 during a workup for multiple myeloma. Subsequent investigation of this mitral valve mass included serial transesophageal echocardiograms (TEEs) and blood cultures, which were all unrevealing and unchanged. The patient had repeat TEEs in August 2018 and December 2019 along with 3 repeat transthoracic echocardiograms during the time span. The partially mobile mass measured approximately 9 mm by 6 mm between the P2 and P3 leaflets (Figure 1, Supplemental Video 1), with stable measurements in all further imaging studies. The patient elected for conservative management given the lack of symptoms and stable position and size of the mass. Prior to WATCHMAN insertion, the authors discussed risk of possible dislodgment and embolization of the mitral valve mass during the procedure, given its mobility and proximity to the leaflet tip seen on TEE (Figure 1, Supplemental Video 1).

Using a multidisciplinary approach with interventional cardiology, we planned to place a temporary cerebral protection device (SENTINEL) prophylactically during the procedure and prior to WATCHMAN implantation in the event of mitral valve mass embolization.

### Management: Placement of the SENTINEL and WATCHMAN device

The patient was prepped in the usual sterile fashion. A TEE was performed after induction of anesthesia, which verified the lack of an LAA thrombus, lack of pericardial effusion, and the continued presence of the chronic mitral valve mass that remained unchanged when compared to prior echo studies. The LAA orifice measured a maximum of 22 mm in diameter at the 135^o^ orientation.

A prior magnetic resonance angiography of the neck revealed a normal aortic arch and normal-caliber brachiocephalic and left carotid arteries. Then, the right radial artery was accessed using the modified Seldinger technique, and a 6F GlideSheath Slender sheath (Terumo Medical Corporation, Somerset, NJ) was inserted into the vessel. Next, the SENTINEL cerebral protection device was advanced to the aortic arch over a 0.014 mm Choice PT wire (Boston Scientific, Marlborough, MA). The device was deployed in the brachiocephalic and left carotid arteries according to the standardized protocol (Supplemental Video 2).

Next, attention was turned to the WATCHMAN implantation procedure. Femoral venous access was obtained, and a heparin bolus was given and an activated clotting time of >250 seconds was confirmed prior to transseptal puncture. A standard 8.5F SL1 sheath (St. Jude Medical, St. Paul, MN) with a radiofrequency-powered NRG transseptal needle (Baylis Medical, Toronto, ON, Canada) was used, which was uneventful. A 14F double-curve WATCHMAN access sheath was carefully introduced to the left atrium with continuous TEE monitoring. With the help of the TEE team and biplane imaging, we attempted to keep the mitral valve mass localized throughout the case and took careful effort to avoid contacting that area with any catheters or wires, by keeping them posterior to the mitral valve. A 27 mm WATCHMAN (Version 2.5) device was successfully deployed in the LAA after a single deployment requiring no partial or full recaptures.

**Figure 2:**
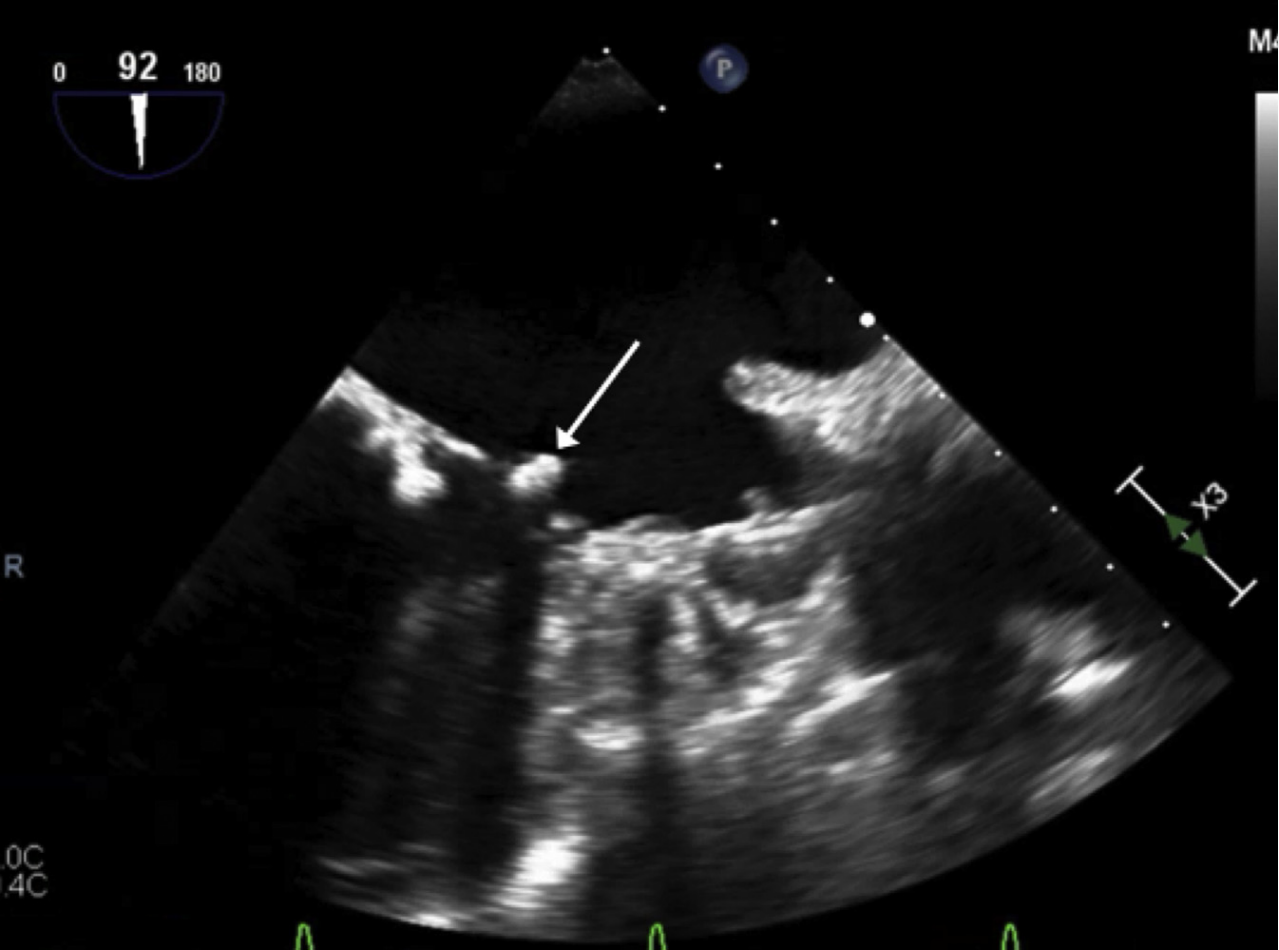
Intraoperative image of mitral valve mass adjacent to the WATCHMAN (Boston Scientific, Marlborough, MA) device. Transesophageal echocardiogram image at 90^o^ demonstrating the proximity of a mitral valve mass (*white arrow*) to the deployed WATCHMAN device.

At the end of the procedure, the SENTINEL device was successfully retracted through the sheath via right radial arterial access. No debris or thrombi were noted to be collected within the SENTINEL device. Successful closure of the access sites was subsequently performed without immediate bleeding complications. Postprocedural TEE imaging revealed no change to the chronic mitral valve mass, which was still present in the same form at the end of the procedure (Figure 2, Supplemental Video 3). The patient was extubated and awoke from anesthesia without any neurologic deficits or evidence of systemic thromboembolism.

## Discussion

Cardioembolic protection devices have been used with success during WATCHMAN implantation in prior series, but none with a known mitral valve mass. Most of these patients demonstrated incidental embolic debris collected in the filter post-implant.^9^ Tan and colleagues^10^ have also described successful placement of WATCHMAN and SENTINEL devices in 2 patients with known chronic LAA thrombus. We describe a unique case of a SENTINEL device prophylactically used to prevent debris embolization in a patient with a known mitral valve mass during LAAO. The potential neurological benefit gained from the SENTINEL device during TAVR is under investigation but is promising.^6^^,^^7^ Knowing that debris dislodgment is also possible during WATCHMAN insertion, we hypothesized the same cerebral protection could feasibly be provided to our higher-risk patient. A limitation of the SENTINEL device is that the left vertebral artery, which branches from the left subclavian artery, remains unprotected to debris embolization.

In addition, we demonstrate that WATCHMAN insertion can be safely performed in a patient with a chronic mitral valve mass, where risk of mass or debris embolization may exist. While thrombus within the LAA is a contraindication to WATCHMAN insertion, masses elsewhere in the left atrium can be considered on a case-by-case basis. Further investigation of cardioembolic protection devices prior to WATCHMAN insertion are needed to better understand the potential long-term neurological benefit from use of these devices.

### Follow-up

**Figure 3:**
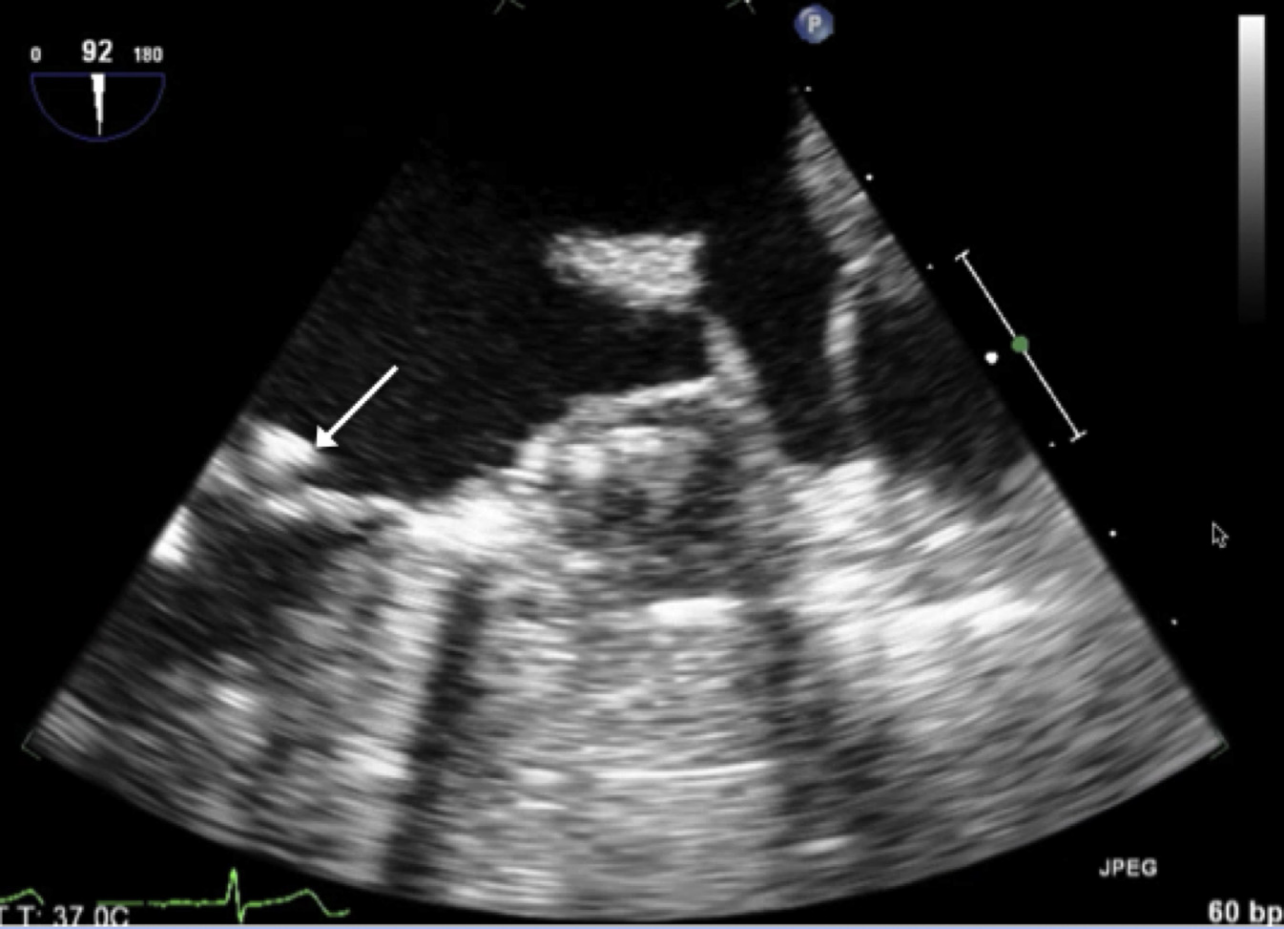
A 45-day post-procedural transesophageal echocardiogram (TEE) image demonstrating the mitral valve mass and WATCHMAN (Boston Scientific, Marlborough, MA) device. TEE image at 90^o^ confirming a stable mitral valve mass (*white arrow*) and endothelialized WATCHMAN device.

The patient had a normal postprocedure course. His oral anticoagulation was discontinued after a 45-day postprocedure TEE demonstrated no evidence of leak or thrombus. The TEE also revealed the unchanged mitral valve mass (Figure 3 and Supplemental Video 4). His aspirin and clopidogrel were continued for another 4½ months, after which he remained solely on aspirin. He has had close follow-up without any neurological issues since the procedure.

## Conclusion

Chronic mitral valve masses are uncommon. We report the first use a of prophylactic SENTINEL device for cerebral protection to prevent cardioembolic stroke during the same procedure and prior to WATCHMAN insertion. This technique may be considered to prevent strokes in patients with chronic cardiac masses who otherwise meet criteria for LAA occlusion device.

**Key Teaching Points**

• Cardioembolic stroke risk can be mitigated with prophylactic placement of a SENTINEL device (Boston Scientific, Santa Rosa, CA) for cerebral protection in select patients.
• The WATCHMAN (Boston Scientific, Marlborough, MA) left atrial appendage (LAA) occlusion device can safely be deployed in patients with chronic mitral valve masses.
• Chronic cardiac masses should not exclude consideration for LAA occlusion in select patients, with the advent of cerebral protection devices.

## Appendix Supplementary data

Supplemental Video 1 Visualization of a mitral valve mass prior to procedure*.* TEE clip at 90^o^ of a mitral valve mass (white arrow) adjacent to the LAA.

Supplemental Video 2 SENTINEL device deployment under fluoroscopy via a right radial arterial approach. Cine clip of the SENTINEL device (white arrows) in the appropriate position within the brachiocephalic and left carotid arteries.

Supplemental Video 3 Intra-operative clip of mitral valve mass and WATCHMAN device. TEE video at 90^o^ demonstrating the proximity of a mitral valve mass (white arrow) to the WATCHMAN device within the LAA.

Supplemental Video 4 45-day post-procedural video demonstrating a mitral valve mass and WATCHMAN device. TEE image at 90^o^ confirming a stable mitral valve mass (white arrow) and endothelialized WATCHMAN device.
